# Merkel cell carcinoma in lymph nodes with and without primary origin

**DOI:** 10.1002/cam4.4562

**Published:** 2022-02-06

**Authors:** Shlomit Fennig, Yosef Landman, Ronen Brenner, Salem Billan, Eyal Fenig

**Affiliations:** ^1^ Institute of Oncology, Edith Wolfson Medical Center Holon Israel; ^2^ Sackler Faculty of Medicine Tel Aviv University Tel Aviv Israel; ^3^ Institute of Oncology, Davidoff Center, Rabin Medical Center, Beilinson Hospital Petah Tikva Israel; ^4^ Division of Oncology Rambam Health Care Campus Haifa Israel; ^5^ Ruth & Bruce Rappaport Faculty of Medicine Technion – Israel Institute of Technology Haifa Israel

## Abstract

The prognosis of MCC with lymph node involvement was better in patients with an unknown than a known primary. Treatment with a uniform aggressive combined chemoradiation regimen, with or without lymphadenectomy, led to better survival rates than previously reported.

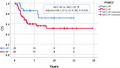

## Abstract

### Background

Merkel cell carcinoma (MCC) tends to spread by lymphatic and hematogenous pathways. Lymph node involvement predicts poor prognosis. This study sought to compare outcome between patients with lymph node involvement and an unknown primary (MCC‐UP) or a primary tumor in skin (MCC‐KP) and to investigate the effectiveness of our aggressive combined chemoradiation regimen.

### Methods

The cohort included 29 patients with MCC‐UP and 43 with MCC‐KP attending a tertiary medical center in 1984–2018 who were treated with a uniform chemoradiation regimen including cis‐platinol or carboplatin and etoposide. Median follow‐up time was 75 and 83 months, respectively. Overall survival (OS) and progression‐free survival (PFS) were compared between the groups.

### Results

Kaplan–Meier analysis showed that compared to the MCC‐KP group the MCC‐UP group had a significantly higher 5‐year PFS (79% vs. 48.8%, *p* = 0.019) and OS (85.3% vs. 59.3%, *p* = 0.02). On multivariate Cox proportional hazard regression analysis including age, sex, lymphatic region (neck/axilla/groin), surgery to the lymph node basin (dissection/biopsy only), and primary status (known/unknown), only primary status had a significant effect on PFS [HR 2.9 (1.2–7.1), *p* = 0.019] and OS [HR 3.4 (1.2–9.4), *p* = 0.020]. In the MCC‐UP group, there were no significant differences in survival between patients (*n* = 15) treated with definitive chemoradiation only and patients (*n* = 14) treated with radical surgery followed by chemoradiation.

### Conclusions

MCC‐UP is associated with a significantly better prognosis than MCC‐KP. Survival rates exceeded those reported for stage III MCC, possibly owing to the aggressive combined chemoradiotherapy regimen. Further research on the use of checkpoint inhibitors is warranted.

## 1 INTRODUCTION

Merkel cell carcinoma (MCC) is a rare aggressive neuroendocrine tumor of skin origin first described in 1972.^1^ MCC tends to spread by lymphatic and hematogenous pathways, and prognosis is mainly impacted by tumor size, presence of nodal metastases, and presence of distant metastasis.

In rare cases, patients present with clinically positive nodal disease and characteristic immunohistochemical findings of MCC in the absence of an identifiable primary tumor. The reported rate of MCC of unknown primary tumor origin (MCC‐UP) in studies of large cohorts ranges between 8% and 19%.^2^, ^3^, ^4^ Based on an analysis of 5823 cases of MCC,^5^ the 7th edition of the American Joint Committee on Cancer Staging Manual, published in 2010,^6^ differentiated detectable microscopic node‐positive MCC (stage IIIA) from clinically detectable macroscopic node‐positive MCC (stage IIIB). Thus, by definition, MCC‐UP was categorized as stage IIIB. However, in 2016, analysis of 9387 cases of MCC showed that MCC‐UP (*n* = 336) was associated with better prognosis than MCC with lymph node involvement and a known primary at presentation (MCC‐KP) (overall survival [OS] estimates, 42% vs. 27%).^2^ This observation supported findings of several earlier case series^7^, ^8^, ^9^, ^10^, ^11^ and was incorporated in the 8th edition of the AJCC Staging Manual in which MCC‐UP was recategorized as stage IIIA.

However, it remains unclear whether malignant cells in MCC‐UP arise de novo from neural cells located in the involved lymph nodes or if the primary tumor undergoes spontaneous regression after spreading to the regional lymph nodes. There are case reports describing spontaneous regression of primary MCC.^12^, ^13^, ^14^, ^15^, ^16^ Findings of tumor‐infiltrating lymphocytes suggested that regression of the primary lesion may be attributable to immune‐mediated mechanisms.^16^ Immunotherapy with checkpoint inhibitors (CPI) have shown remarkable and durable results in metastatic disease.^17^, ^18^, ^19^ Ongoing research in the neoadjuvant and adjuvant settings are pending, with case reports supporting the use of CPI in earlier stages.^20^

The existing case series of MCC‐UP were generally small and heterogeneous,^7^, ^8^, ^9^, ^10^, ^11^ and the outcome of this patient group warrants further research. Furthermore, owing to the rarity of the tumor, there is no consensus regarding the optimal treatment of patients with stage III MCC, particularly MCC‐UP. The aim of the present study was to compare prognosis between patients with MCC‐UP and MCC‐KP and to investigate the effectiveness of our aggressive combined chemoradiation treatment regimen, with or without lymphadenectomy.

## 2 PATIENTS AND METHODS

### 2.1 Patients and setting

The study conforms to the standards required by the Declaration of Helsinki and was approved by the Institutional Review Board of Rabin Medical Center (0101‐07)which waived the need for informed consent. A retrospective design was used. The electronic healthcare database of a tertiary university‐affiliated medical center was searched for patients diagnosed with MCC in 1984–2018. Staging was based on clinical examination, computed tomography, positron emission tomography, or their combination. Patients with lymph node involvement (stage IIIA, IIIB, AJCC 8th edition) were identified, and their demographic and clinical data were collected. The cohort was then divided into two groups for comparison: unknown cutaneous primary origin (MCC‐UP) and known cutaneous primary tumor (MCC‐KP).

### 2.2 Treatment

For the treatment of MCC, our department uses a multimodality approach consisting of chemoradiation with or without surgery, as described in our previous publications.^21^, ^22^ The chemotherapy regimen includes cis‐platinol 20 mg/msq on days 1–5 with etoposide 100 mg/msq on days 1, 3, 5, every 28 days for four to six cycles. The first 2 cycles are given concomitantly with radiotherapy. The total radiation dose is 45–50 Gy delivered in 25 fractions of 1.8–2 Gy each to the primary and involved lymphatic field with a sequential boost of 9–10 Gy is delivered to the macroscopic disease. We used three‐dimentional conformal radiotherapy (3‐DCRT) or intensity modulated radiotherapy using modern linear accelerators (Varian).

### 2.3 Outcome measures

The primary outcome measures of the study were progression‐free and OS.

### 2.4 Statistical analysis

The MCC‐UP and MCC‐KP groups were compared for demographic and clinical characteristics using Student *t*‐test for continuous parameters and chi‐square test for categorical parameters. Progression‐free survival (PFS) and OS were calculated from the date of diagnosis to the date of last follow‐up or death. OS and PFS were evaluated with the Kaplan–Meier curve and compared between the groups using cox proportional hazard regression model. Univariate and Multivariate analyses were performed including all demographic and clinical variables that could potentially impact survival. All statistical analyses were done using SPSS software (IBM Corp., 2017. IBM SPSS Statistics for Windows).

## 3 RESULTS

Of the 198 patients diagnosed with MCC during the study period, 72 (36%) had regional lymph node metastases. They included 29 patients (14% of MCC patients) with MCC‐UP and 43 (22% of MCC patients) with MCC‐KP. The median duration of follow‐up was 75 months (range 13–192) in the MCC‐UP group and 83 months (12–287) in the MCC‐KP group. Most of the patients in both groups were male; average age was 69.0 years in the MCC‐UP group and 72.5 years in the MCC‐KP group (*p* = 0.46).

The most common site of lymph node involvement was the groin in the MCC‐UP group 18/29(59%) and the head and neck in the MCC‐KP group 18/43(42%). The most common primary skin tumor in the MCC‐KP group was identified in the face (scalp region). In 12 patients with a primary skin lesion (24%), lymph node involvement was first discovered by sentinel lymph node biopsy.

Most of the patients in both groups were immunocompetent. One woman in the MCC‐UP group was under immunosuppression drugs following kidney transplantation. In the MCC‐KP group, one patient was under antiviral maintenance due to infection with human immunodeficiency virus and another was after chemotherapy for lymphoma (Table 1).

Chemoradiation was preceded by radical lymphadenectomy in 14 patients in the MCC‐UP group and 34 patients in the MCC‐KP group. The remainder (*n* = 15 and *n* = 9, respectively) underwent biopsy only. The same chemoradiation regimen was used in both groups, with higher doses of radiation in the patients who were not operated. The main late effect seen after treatment was lymphedema, which was observed only in patients who underwent complete lymph basin dissection in the axilla or groin, occurring in 23/72(32%) of our cohort.

Recurrence was observed in 27 patients, within 18 months from diagnosis in all cases and during the first year in most (93%). Recurrence was lower in the MCC‐UP group than the MCC‐KP group (5/29, 17.2% vs. 24/43, 55.8%, *p* < 0.002). All 5 patients in the MCC‐UP group had distant recurrence. Two are currently in complete remission under treatment with avelumab, an anti‐PDL1 inhibitor. The other three died. In the MCC‐KP group, 19 patients had distant recurrence and five regional recurrences. One patient, with local‐regional recurrence was salvaged by repeated chemoradiation and is still in remission at 43 months, and one patient is currently in unmaintained remission following avelumab treatment. The remaining 22 patients died of the disease.

**FIGURE 1:**
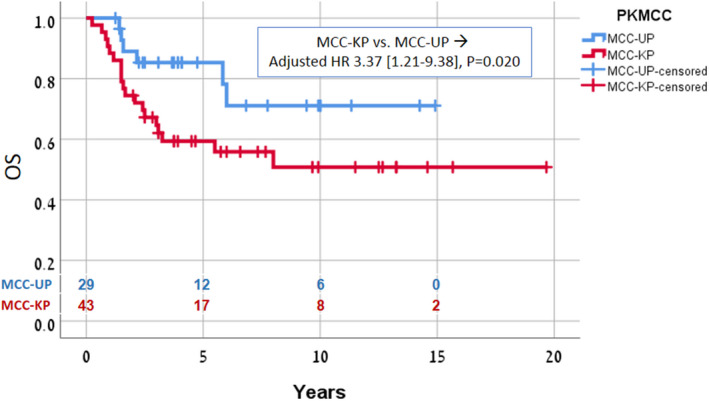
Comparison of OS between MCC‐UP and MCC‐KP groups. OS, overall survival; MCC‐KP, Merkel cell carcinoma with known primary; MCC‐UP, Merkel cell carcinoma of unknown primary tumor origin

**FIGURE 2:**
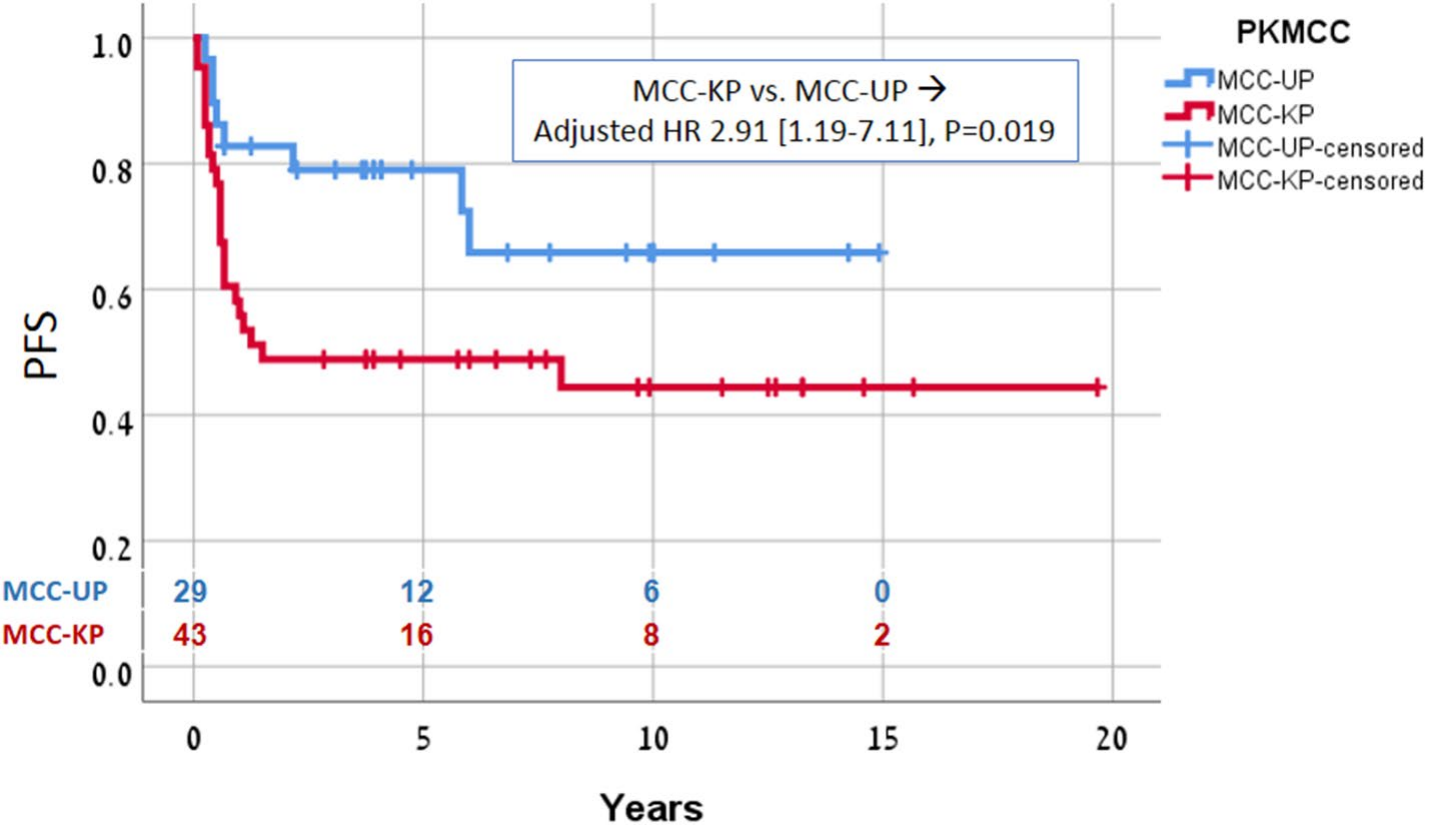
Comparison of PFS between MCC‐UP and MCC‐KP groups. PFS, progression‐free survival; MCC‐KP, Merkel cell carcinoma with known primary; MCC‐UP, Merkel cell carcinoma of unknown primary tumor origin

For the whole cohort with stage III MCC, 5‐year OS and PFS were 69.4% and 60.7%, respectively. Compared to MCC‐KP, MCC‐UP was associated with a significantly higher 5‐year PFS (79% vs. 48.8%, *p* = 0.019) and higher OS (85.3% vs. 59.3%, *p* = 0.020) (Figures 1 and 2). On complete multivariate Cox proportional hazard regression analysis including age, sex, lymphatic region (neck, axilla, or groin), surgery to lymph node basin (dissection or biopsy only), and primary status (known or unknown), only primary status had a significant effect on PFS [HR 2.9 (1.2–7.1), *p* = 0.019] and OS [HR 3.4 (1.2–9.4), *p* = 0.02) (Table 2). While omitting extensive surgery was associated with a trend for lower OS [HR 2.31 (0.9–5.7), *p* = 0.07], in the MCC‐UP group, there were no significant differences in PFS and OS between patients treated with definitive chemoradiation to grossly involved lymph nodes and patients treated with radical surgery and adjuvant chemoradiation.

**TABLE 1:** Characteristics of patients with stage III MCC

Characteristic	MCC‐UP (*n* = 29)	MCC‐KP (*n* = 43)	*p* value
Male gender	21 (72%)	33 (77%)	NS
Ashkenazi Jewish origin	21 (72%)	38 (81%)	NS
Age (years), mean (range)	69.0 (54–86)	72.5 (48–89)	NS
Lymph node sites			
Head & neck	6 (21%)	18 (42%)	0.04
Axilla	5 (20%)	11 (26%)	NS
Groin	18 (59%)	14 (32%)	0.02
Immunosuppression	1 (4%)	2 (4%)	NS

*Note*: Data are presented as *n* (%) unless otherwise stated.

Abbreviations: MCC‐KP, Merkel cell carcinoma with known primary; MCC‐UP, Merkel cell carcinoma of unknown primary tumor origin.

**TABLE 2:** Multivariate Cox regression survival analysis

Characteristic	HR	95.0% CI for HR		*p* value	HR	95.0% CI for HR		*p* value
		Lower	Upper			Lower	Upper	
	Multivariate PFS analysis				Multivariate OS analysis			
Age (years)	1.02	0.99	1.06	0.146	1.02	0.98	1.05	0.281
Sex	0.70	0.30	1.59	0.391	0.51	0.19	1.38	0.186
Region								
Groin vs. H&N	0.99	0.42	2.33	0.98	0.77	0.30	2.00	0.60
Axilla vs. H&N	0.86	0.33	2.22	0.75	0.61	0.21	1.78	0.37
Surgery to lymphatic basin Biopsy vs. Dissection	1.93	0.86	4.32	0.109	2.31	0.93	5.70	0.070
Primary status MCC‐KP vs. MCC‐UP	2.91	1.19	7.11	0.019	3.37	1.21	9.38	0.020

Abbreviations: HR, hazard ratio; CI, confidence interval, PFS, progression‐free survival; OS, overall survival; H&N, head and neck; MCC‐KP, Merkel cell carcinoma with known primary; MCC‐UP, Merkel cell carcinoma of unknown primary tumor origin.

**TABLE 3:** Literature review of patients with stage III MCC

Author	No. pts. with MCC‐UP	Recurrence	5‐year PFS (%)	5‐year OS (%)
Deneve et al.^7^	38	13 (34%)	50	74
Foote et al.^8^	36		61	75
Tarantola et al.^9^	23	8 (35%)		63
Chen et al.^10^	16	4 (25%)		
Vandeven et al.^11^				66
Present study	29	5 (17%)	79	85

*Note*: Data are presented as *n* (%).

Abbreviations: PFS, progression‐free survival; OS, overall survival; MCC‐UP, Merkel cell carcinoma of unknown primary tumor origin.

## 4 DISCUSSION

We present a large series of patients with MCC‐UP with the longest reported follow‐up to date. Our findings demonstrated that patients with MCC‐UP treated by chemoradiation, with or without lymphadenectomy, have an excellent prognosis. Both OS and PFS were significantly better than in the patients presenting with a cutaneous primary with regional lymph node involvement (MCC‐KP) given similar treatment (Figures 1 and 2). This observation is in line with several earlier case series^7^, ^8^, ^9^, ^10^, ^11^ and a recent analysis of a large cohort of patients with MCC.^2^ It is further supported by our use of the same combined chemoradiation regimen in all patients whereas the earlier studies used heterogeneous treatment approaches.

Moreover, the aggressiveness of our treatment regimen may explain the higher rates of PFS and the lower rates of recurrence in our cohort compared to those reported in the earlier case series (Table 3).^7^, ^8^, ^9^, ^10^, ^11^ It is noteworthy that OS and PFS in the MCC‐KP group were also better than reported in the SEER data^23^ and the recent large cohort study.^2^ The curative potential of our chemoradiation regimen is further indicated by the successful treatment of 11 of the 15 patients in the MCC‐UP group (73%) who had advanced unresectable or borderline‐resectable tumors.

MCC tumor is considered highly immunogenic. Studies have shown that in 80% of cases, Merkel polyomavirus is clonally integrated and induces persistent immunogenicity,^24^ while the remaining 20% of virus‐negative patients are highly exposed to ultraviolet radiation, resulting in a high mutational burden that is associated with tumor immunogenicity.^25^ Thus, the better prognosis of patients with MCC‐UP than patients with MCC‐KP may be explained by their better inherent immune system competence. This hypothesis is in line with the assumption that the regression of the primary lesion is attributable to immune‐mediated mechanisms.^8^, ^9^, ^10^ Accordingly, a recent study^10^ demonstrated higher levels of tumor‐specific antibodies and a higher tumor mutational burden in patients with MCC‐UP, suggesting enhanced tumor immunogenicity and immune‐mediated clearance of primary skin lesions. It is noteworthy that in our cohort, the comorbid immunosuppression factors were evenly distributed between patients with MCC‐UP and MCC‐KP, ruling them out as potential contributors to the between‐group difference in prognosis. Furthermore, multivariate analysis yielded no other factors, demographic or clinical, that could account for the better prognosis in the MCC‐UP group.

In conclusion, our study demonstrated a better prognosis of patients with MCC‐UP than patients with MCC‐KP, in agreement with earlier studies. Our results were improved over those reported previously, which might be explained by our unique use of a uniform, aggressive chemoradiation treatment regimen.

Given the remarkable response shown to CPI in metastatic MCC,^17^, ^18^, ^19^, ^20^ as well as two of the five MCC‐UP patients with disease recurrence, it may be worthwhile, in the future, to incorporate immunotherapy into the treatment of stage III MCC together with the other modalities. Further studies of this issue are warranted.

## CONFLICT OF INTEREST

The authors have no conflicts of interest to declare.

## AUTHOR CONTRIBUTIONS

*Conceptualization, data curation, formal analysis, investigation, methodology, resources, software, writing—original draft, and writing—review and editing*: Shlomit Fennig. *Data curation, formal analysis, methodology, software, and writing—review and editing*: Yosef Landman. *Data curation, methodology, and writing—review and editing*: Ronen Brenner. *Data curation, methodology, and writing—review and editing*: Salem Billan. *Supervision, validation, visualization, writing—original draft, and writing—review and editing*: Eyal Fenig.

## Data Availability

The data that support the findings of this study are available from the corresponding author upon reasonable request.
